# Breaking the silence: a cross-sectional study of psychological capital, job embeddedness, and silence behavior in operating room nurses

**DOI:** 10.3389/fmed.2026.1772558

**Published:** 2026-07-01

**Authors:** Jinfeng Qi, Yu Zhang, Qiaomei Cheng, Shuai Wang, Zhi Zou

**Affiliations:** 1 Henan Provincial People’s Hospital, Zhengzhou, China; 2 Henan Provincial Chest Hospital, Zhengzhou, China; 3 988 Hospital of Joint Logistics Support Force, Henan, China

**Keywords:** job embeddedness, nurses, operating room, psychological capital (PC), silence behavior

## Abstract

**Objective:**

This study aimed to understand the current status of silence behavior, psychological capital, and job embeddedness among operating room (OR) nurses, as well as to explore the relationships among these factors.

**Methods:**

A cross-sectional study design was employed to investigate the relationships among psychological capital, job embeddedness, and silence behavior among OR nurses. Participants from four hospitals in Henan Province, China, were recruited via convenience sampling between July 2022 and October 2022. The study utilized the General Socio-Demographic Characteristics questionnaire, the Psychological Capital Scale, the Job Embeddedness Scale, and the Silence Behavior Scale. Structural equation modeling was performed using SPSS version 25.0 and AMOS version 26.0 to analyze the interrelationships among these factors.

**Results:**

A total of 395 nurses participated in the study, with an average age of 32.68 years (± 6.17). Among the participants, 143 were male (36.2%) and 252 were female (63.8%). The overall scores for psychological capital, job embeddedness, and silent behavior were found to be at a moderate level. The silent behavior of OR nurses exhibited a negative correlation with psychological capital. Job embeddedness showed a significant negative correlation with silent behavior, while it was positively correlated with psychological capital. Additionally, job embeddedness played a partial mediating role in the relationship between psychological capital and silence behavior among OR nurses.

**Conclusion:**

The levels of silence behavior, psychological capital, and job embeddedness among OR nurses were moderate. It is suggested that managers may help improve the existing conditions of silent behavior by attending to consider attending to the psychological capital and job embeddedness of OR nurses, which were associated with lower levels of silence behavior in this study.

## Introduction

The operating room (OR) is a highly specialized and high-risk clinical setting in which nurses play an indispensable role in maintaining the continuity, coordination, and safety of surgical care (1). Beyond assisting in routine procedures, OR nurses frequently respond to emergency surgeries, placing extreme demands on their professional competence, adaptability, and physical and mental resilience (2). Characterized by high intensity, heavy workload, time pressure, elevated occupational exposure, and prolonged mental strain, work in the OR often leaves nurses in a persistent state of stress and fatigue. Compared with nurses in other departments, OR nurses are more prone to emotional suppression, increased psychological burden, and hesitation in communication—conditions that may co-occur with workplace silence behavior (3).

Effective communication is essential for ensuring patient safety, fostering teamwork, and driving quality improvement in healthcare organizations, particularly in surgical contexts requiring rapid decision-making and multidisciplinary collaboration (4). Nevertheless, not all nurses who identify potential problems, risks, or opportunities for improvement are willing to speak up proactively (5). Some nurses deliberately withhold their concerns, suggestions, or professional judgments even when aware of their clinical value. This phenomenon is commonly conceptualized as silence behavior (6). The “silence effect,” first documented in 1970, describes the psychological discomfort employees experience when communicating information that leaders prefer to ignore, leading them to avoid reporting relevant issues or remain entirely silent (7). Morrison and colleagues later expanded this concept and defined organizational silence as a collective phenomenon in which employees intentionally withhold opinions, concerns, or recommendations regarding organizational problems (6). Within nursing, silence behavior is further defined as the act by which nurses conceal, filter, or fail to express work-related opinions and suggestions that could improve practice based on their professional expertise, due to organizational, interpersonal, or individual constraints (8).

References to the literature indicate that the study of silence behavior originated in management science and has since been widely applied in healthcare. Previous studies have employed diverse methodologies to investigate silence behavior, including descriptive surveys (9), correlational analyses (10, 11), and theoretical investigations (12, 13). Since Zheng et al. formally defined silence behavior in the Chinese context in 2008 (14), research on nurse silence has grown steadily. Available evidence indicates that nurse silence is shaped by interacting factors at individual, social, and organizational levels. At the individual level, relevant factors include demographic, physiological, and psychological characteristics. Demographic and physiological attributes include gender, age, clinical experience, and educational level (15); psychological factors include negative emotions (16), work engagement (17), and job satisfaction (18). Social factors mainly involve the quality of teamwork (19) and ethnic or cultural influences (20). Within Chinese cultural contexts, researchers have noted that employee silence may also be affected by collectivism, individualism, concepts of “face” and “renqing (interpersonal favor),” Confucian traditions, and power distance. At the organizational level, managerial style (21) and perceived organizational justice (22) are particularly critical. Prior scholars have demonstrated that features of transformational leadership—such as openness, inclusiveness, innovativeness, and willingness to listen—reduce silence among OR nurses, whereas authoritarian leadership and abusive supervision reinforce such behavior (23).

Silence behavior has emerged as a major concern in organizational development and clinical management, as it can generate adverse consequences for both healthcare providers and patients. In surgical settings, silence among OR nurses may be associated with delayed reporting of safety risks, impair team communication, compromise decision-making quality, and ultimately undermine nursing care quality and surgical outcomes (22). Despite its clinical importance, research specifically targeting silence behavior among OR nurses remains limited. In particular, although scholars have explored determinants of nurse silence from multiple perspectives, relatively little attention has been paid to the roles of positive psychological resources and work-related bonding in explaining why OR nurses choose to remain silent.

Psychological capital and job embeddedness may provide a valuable theoretical framework for understanding silence behavior among OR nurses. Psychological capital, a core construct within positive organizational behavior, was formally conceptualized by Luthans and colleagues as an individual’s positive psychological state of development characterized by four interrelated components: self-efficacy (confidence in exerting the necessary effort to succeed at a given task), hope (persevering toward goals and redirecting pathways when necessary), resilience (bouncing back and beyond from adversity), and optimism (positive attributions about current and future success) (24). Grounded in Conservation of Resources (COR) theory (25), psychological capital is conceptualized as a higher-order personal resource that enables individuals to accumulate, protect, and deploy other resources in demanding work contexts. Employees with greater psychological resources may be better positioned to cope with work stressors and to engage in proactive communicative behaviors rather than retreating into self-protective strategies such as silence. Accordingly, nurses with higher psychological capital are expected to express concerns with greater confidence, sustain resilience in the face of interpersonal risk, and approach organizational challenges with optimism and agency—characteristics that may be associated with lower levels of silence behavior.

Job embeddedness, originally developed by Mitchell and colleagues (26), is a multidimensional construct capturing the extent to which employees are embedded in their jobs and communities through three interrelated mechanisms: links (formal or informal connections between individuals and institutions or people), fit (perceived compatibility between an individual and their organization or community), and sacrifice (the perceived material and psychological costs associated with leaving one’s job). Each mechanism operates at both the organizational and community levels, yielding six subdimensions. Grounded in Social Exchange Theory (27), higher job embeddedness reflects stronger relational bonds, greater perceived compatibility, and elevated perceived costs of exit, which may be associated with reciprocal investment in organizational welfare. For OR nurses, greater embeddedness implies closer identification with the professional community, stronger interpersonal bonds, and heightened awareness of the costs of withdrawal—all of which may correspond to a lower threshold for voicing concerns and, in turn, less frequent silence behavior.

The theoretical rationale for incorporating both constructs into the present structural equation model is grounded in their conceptual and empirical complementarity. From the perspective of COR theory, psychological capital may be regarded as an internal resource that is plausibly associated with deeper organizational embeddedness as it may relate to “nurses” capacity to form stronger interpersonal connections, perceive greater person–organization fit, and better tolerate the sacrifices associated with long-term organizational commitment. In turn, drawing on Social Exchange Theory, greater embeddedness may be associated with a sense of reciprocal obligation to the organization, which is in turn linked to more proactive contributions—including the expression of professional concerns rather than remaining silent. This logic supports a theoretically coherent indirect pathway in which the association between psychological capital and lower silence behavior is partly accounted for by job embeddedness, which constitutes the central mediating hypothesis of the present study.

Although a considerable body of research has examined pairwise relationships among psychological capital, job embeddedness, and silence behavior (23), few studies have systematically explored their interrelationships among OR nurses as a distinct population. Existing evidence preliminarily supports a direct association between psychological capital and silence behavior, yet the underlying pathway remains insufficiently clarified. In particular, whether job embeddedness plays a mediating role in the relationship between psychological capital and silence behavior among OR nurses remains largely unexplored. Examining this pathway will deepen understanding of how intra-individual psychological resources and work-related bonding are jointly associated with silence behavior in high-risk nursing environments.

To address the aforementioned gaps, the present study aimed to investigate the current status of silence behavior, psychological capital, and job embeddedness among OR nurses, and to clarify the structural relationships among these three constructs. Specifically, this study pursued the following four objectives: (1) to describe the levels of silence behavior, psychological capital, and job embeddedness in a sample of Chinese OR nurses; (2) to examine the bivariate associations among silence behavior, psychological capital, and job embeddedness; (3) to identify demographic and work-related factors associated with silence behavior; and (4) to test, using structural equation modeling, whether job embeddedness plays a mediating role in the relationship between psychological capital and silence behavior. By examining both the direct association of psychological capital with silence behavior and the indirect pathway through job embeddedness, this study seeks to extend current understanding of the psychosocial correlates of nurse silence in high-risk surgical settings. The findings are expected to provide an empirical and theoretical basis for the development of targeted managerial strategies aimed at supporting “nurses” positive psychological resources and organizational embeddedness, which may in turn be associated with lower levels of silence behavior and with more open communication, effective teamwork, and patient safety in the OR.

Before the research, this study proposed four hypotheses as follows:

(1) The psychological capital of OR nurses exhibits a negative correlation with silent behavior.

(2) The psychological capital of OR nurses demonstrates a positive correlation with job embeddedness.

(3) The job embeddedness of OR nurses is negatively correlated with silent behavior.

(4) Job embeddedness plays a mediating role in the relationship between psychological capital and silent behavior among OR nurses.

## Materials and methods

### Study design and participants

This cross-sectional study was conducted among OR nurses from four Grade III-A hospitals in Henan Province, China, between July and October 2022. Eligible participants were required to meet all of the following inclusion criteria: (1) being a registered nurse actively employed in the OR; (2) having at least 1 year of OR working experience; (3) being engaged in frontline clinical nursing work, and not holding a managerial position (e.g., head nurse) or serving as an assistant nurse; (4) being on-duty during the data collection period (i.e., not temporarily absent due to further education, sick or maternity leave, or pandemic-related restrictions); and (5) providing written informed consent to participate. The exclusion criteria, applied during the course of the study, were: (1) refusal to complete the questionnaires; and (2) refusal to continue cooperating with the researcher.

The required sample size was estimated using Kendall’s rule of thumb (28), which recommends a sample size of 5–10 times the number of items in the largest measurement instrument used. Among the three scales adopted in the present study, the Job Embeddedness Scale was the most comprehensive instrument with 37 items, indicating a minimum required sample of 5 × 37 = 185 and a preferred sample of 10 × 37 = 370 participants. To ensure adequate statistical power for the planned structural equation modeling, this estimate was further benchmarked against Kline’s recommendation (29) of a minimum of 200 cases with a case-to-parameter ratio of at least 10:1 for SEM analyses. After inflating the target by approximately 10–15% to account for invalid or incomplete responses, the planned recruitment was set at approximately 410–440 OR nurses. Ultimately, 412 questionnaires were distributed and returned, of which 395 (95.9%) met the eligibility and completeness criteria and were retained for the final analysis, satisfying all of the above sample size criteria.

### Study tools

Before testing the hypothesized structural model, confirmatory factor analysis (CFA) was conducted in AMOS 26.0 using maximum-likelihood estimation to evaluate the construct validity of the three measurement instruments used in this study—the Psychological Capital Questionnaire, the Job Embeddedness Scale, and the Nurse Organization Silence Assessment Questionnaire—in the current sample. Model fit was evaluated using the χ^2^/df ratio (χ^2^/df < 3 indicating good fit, < 5 acceptable), the comparative fit index (CFI) and Tucker–Lewis index (TLI; > 0.90 acceptable), and the root-mean-square error of approximation (RMSEA; < 0.08 acceptable, < 0.05 good) (30). Standardized factor loadings ≥ 0.50 were considered indicative of satisfactory convergent validity. Internal consistency for each scale was assessed using Cronbach’s α coefficient.

#### The General Sociodemographic Characteristics Questionnaire

It incorporates insights from prior research to identify factors that may influence the occurrence of silence behavior among the study subjects. The survey items encompass age, gender, professional title, in-department administrative role (i.e., whether the participant held an informal coordinator-type role—such as team leader, group leader, or shift coordinator—within their frontline nursing duties; formal nursing managerial positions such as head nurse and deputy head nurse were excluded per the inclusion criteria), educational level, marital status, employment method, work tenure in the OR, monthly income, and the number of night shifts per month.

#### Psychological Capital Questionnaire

This questionnaire was developed by the Chinese scholar Zheng et al. (14), and Ding etal. (31) verified its reliability and validity for Chinese nurses based on this scale, which was tailored to the specific working characteristics of Chinese nurses. The questionnaire comprised 24 items grouped into four dimensions: self-efficacy (items 1–6), hope (items 7–12), resilience (items 13–18), and optimism (items 19–24). Responses were recorded on a 6-point Likert scale (1 = strongly disagree to 6 = strongly agree). Three items (13, 20, and 23) were reverse-scored, with higher dimension scores indicating greater psychological capital. Based on the total score, the overall level of psychological capital was classified into three categories obtained by dividing the total possible score range (24–144) into equal tertiles: low (24–64), moderate (65–104), and high (105–144), with higher categories indicating greater psychological capital. The scale demonstrated high internal consistency in this study (Cronbach’s α = 0.927).

Validity evidence. CFA was performed to examine the construct validity of the scale. The hypothesized four-factor model showed acceptable fit to the data (χ^2^/df = 2.74, RMSEA = 0.067, CFI = 0.912, TLI = 0.904), and all standardized factor loadings exceeded 0.50, supporting the construct validity of the scale in the present sample.

#### Job Embeddedness Scale (JES)

The scale was originally developed by Mitchell et al. (26) to evaluate job embeddedness among OR nurses within their departmental environment. The scale consisted of 37 items across six dimensions: organizational fit, community fit, organizational sacrifice, community sacrifice, organizational links, and community links. Items 1–24 employed a 5-point Likert scale (1 = strongly disagree to 5 = strongly agree); items 25–37 were similarly scored, and all items contributed positively to the total score (range: 37–185). Based on the total score, job embeddedness was categorized as low (37–85), moderate (86–133), or high (134–181). In the current study, the scale showed excellent internal consistency (Cronbach’s α = 0.926).

Validity evidence. CFA was conducted to verify the six-factor structure of the scale in the present sample. The measurement model demonstrated marginally acceptable fit (χ^2^/df = 2.86, RMSEA = 0.071, CFI = 0.903, TLI = 0.896), with TLI approaching the conventional 0.90 threshold. The majority of standardized factor loadings exceeded 0.50, providing tenable evidence of construct validity for the six-factor structure.

#### Nurse Organization Silence Assessment Questionnaire

The questionnaire, developed by Yang (32), was used to evaluate silence behavior among OR nurses in clinical settings. The 20-item scale comprised four dimensions: acquiescent silence (6 items), defensive silence (5 items), prosocial silence (5 items), and indifferent silence (4 items). Responses were recorded on a 5-point Likert scale ranging from 1 (never felt) to 5 (always felt), with total scores ranging from 20 to 100. Higher scores indicated more frequent silence behavior. Based on the total score, the overall level of silence behavior was classified into three categories obtained by dividing the total possible score range (20–100) into equal tertiles: low (20–46), moderate (47–73), and high (74–100), with higher categories reflecting more frequent silence behavior. The scale demonstrated excellent reliability in this study (Cronbach’s α = 0.918).

Validity evidence. CFA was used to test the four-factor structure of the scale. The measurement model showed acceptable fit (χ^2^/df = 2.61, RMSEA = 0.064, CFI = 0.918, TLI = 0.909), and all standardized factor loadings exceeded 0.50, supporting the construct validity of the scale.

### Data collection

This study employed an online survey methodology using the Wenjuanxing (an online questionnaire survey tool), with questionnaires distributed via WeChat after obtaining institutional approvals. Prior to distribution, researchers established a WeChat group to explain the study purpose, procedures, and completion guidelines, while the questionnaire preface contained detailed participant information. Specifically, the questionnaire link and QR code were posted in the dedicated WeChat study group, which contained only the eligible OR nurses who had agreed to participate. The questionnaire preface included an electronic informed consent statement that participants were required to actively acknowledge before they could proceed to the survey items; participation was anonymous and voluntary, and participants could withdraw at any time without any negative consequence. Each nurse completed the questionnaire individually on their own smartphone at a time and place of their convenience, outside of working hours whenever possible.

The survey utilized forced-response formatting (requiring all items to be completed) with an average completion time of 15–20 min. During the data collection period, research team members remained available in the WeChat group in a passive, standby capacity exclusively for procedural and technical support—for example, resolving difficulties in accessing the survey link, navigating the platform interface, or submitting the completed questionnaire. Researchers responded only when participants initiated a query; no proactive contact, reminders, or follow-up messages were sent to individual participants during the response period. Importantly, no interpretation of questionnaire items, discussion of response content, or guidance regarding answers was provided at any point. The anonymous design of the survey ensured that researchers could not link any individual WeChat identity to specific questionnaire responses, thereby minimizing the potential for observer effects or social desirability bias. To prevent duplicate submissions, the survey platform was configured to allow only one submission per WeChat account and device. All responses were automatically stored on the Wenjuanxing secure server and were accessible only to the research team in a de-identified form. From 412 initially collected responses, 17 were excluded due to incompleteness, errors, or failure to meet inclusion criteria, resulting in 395 valid questionnaires (95.9% response rate) for final analysis.

### Data analysis

Data entry was performed using Epidata 3.1, with subsequent analyses conducted in SPSS 25.0 and AMOS 26.0. The statistical analyses were conducted in the following sequence. First, descriptive statistics were computed: categorical sociodemographic variables (gender, professional title, in-department administrative role, educational level, marital status, employment type, and monthly income) were described using frequencies and percentages, whereas the continuous total scores of psychological capital, job embeddedness, and silence behavior were expressed as means ± standard deviations. Second, univariate comparisons of the total scores of psychological capital, job embeddedness, and silence behavior (as dependent variables) across sociodemographic subgroups were performed using independent-samples *t*-tests for dichotomous grouping variables (i.e., gender) and one-way analysis of variance (ANOVA) for grouping variables with three or more categories (i.e., age group, professional title, educational level, in-department administrative role, marital status, employment type, work tenure in the OR, monthly income, and number of night shifts per month). Third, Pearson correlation coefficients were calculated among the three continuous total scores—psychological capital, job embeddedness, and silence behavior—to examine their bivariate associations. Fourth, a simultaneous-entry multiple linear regression model was estimated with the silence behavior total score as the dependent variable; the independent variables comprised the psychological capital total score, the job embeddedness total score, and those sociodemographic variables that had reached statistical significance (*p* < 0.05) in the preceding univariate comparisons. To avoid intra-construct redundancy and the multicollinearity that would arise from including highly correlated subdimensions of the same higher-order construct, only the total scores of psychological capital and job embeddedness—and not their subdimensions—were entered as predictors. Multicollinearity among the predictors was then formally assessed using variance inflation factor (VIF) and tolerance statistics. Following commonly accepted criteria, a VIF value below 5 (and ideally below3) combined with a tolerance value above 0.20 was regarded as indicating that multicollinearity was not a serious concern.

For mediation analysis, confirmatory factor analysis (CFA) was first conducted to evaluate the measurement models of the three latent constructs, and structural equation modeling (SEM) was subsequently used to test whether job embeddedness plays a mediating role in the relationship between psychological capital and silence behavior. Model fit was assessed using multiple indices: χ^2^/df ratio (where lower values indicate better fit), RMSEA (< 0.08 acceptable), and Comparative Fit Index (CFI), Adjusted Goodness-of-Fit Index (AGFI), Goodness-of-Fit Index (GFI), Normed Fit Index (NFI), Incremental Fit Index (IFI), and Tucker-Lewis Index (TLI) (range 0–1, with values > 0.90 suggesting good fit). A two-step analytical strategy was adopted for SEM: first, CFA was conducted to evaluate the measurement models of the three latent constructs; second, the structural model was tested after acceptable measurement model fit had been established.

Because all study variables were measured using self-report instruments, the potential influence of common method bias (CMB) was carefully considered, and several procedural and statistical measures were taken to assess and mitigate this risk. At the procedural level, the following controls were implemented during the study design phase: (1) the survey was administered anonymously, reducing the likelihood of socially desirable responding; (2) different response scale formats were used across instruments (a 6-point Likert scale for psychological capital versus 5-point Likert scales for job embeddedness and silence behavior), thereby reducing the likelihood of pattern responding or artifactual covariance due to scale format consistency; and (3) the questionnaire preface did not disclose the hypothesized relationships among the study variables, minimizing demand characteristics. At the statistical level, Harman’s single-factor test was conducted as a *post hoc* procedure to assess the severity of CMB. An exploratory factor analysis including all measurement items was performed using unrotated principal component analysis. The results revealed multiple factors with eigenvalues greater than 1, and the first unrotated factor accounted for 32% of the total variance, which was below the commonly recommended 50% threshold (33), suggesting that CMB was unlikely to pose a serious threat to the validity of the present findings. Nevertheless, these measures cannot entirely eliminate the possibility of CMB, and this limitation should be considered when interpreting the results.

## Results

### Descriptive statistics

The study included 395 OR nurses (36.2% male, 63.8% female) with a mean age of 32.68 ± 6.17 years. Mean total scores indicated moderate levels across all measures: psychological capital (107.66 ± 16.81), job embeddedness (125.71 ± 18.55), and silence behavior (49.31 ± 15.83), shown in Table 1. Among the four dimensions of silence behavior, prosocial silence had the highest item mean score (2.89), followed by defensive silence (2.77), acquiescent silence (2.20), and indifferent silence (1.95). On the 1–5 response scale, the item means of prosocial silence (2.89) and defensive silence (2.77) clearly exceeded the scale midpoint of 2.5, whereas those of acquiescent silence (2.20) and indifferent silence (1.95) fell below this midpoint, indicating that prosocial and defensive silence constituted the more dominant motivational dimensions, while acquiescent and indifferent silence were comparatively less prevalent. The same rank order was observed for the dimension total scores: prosocial silence (14.45 ± 4.50) and defensive silence (13.85 ± 4.30) made the largest contributions to the overall silence behavior score, whereas indifferent silence (7.81 ± 2.95) contributed the least. Notably, although prosocial and defensive silence were close in magnitude, prosocial silence consistently ranked highest across both item-mean and total-score comparisons, suggesting it was the single most prominent form of silence among OR nurses. Taken together, these findings indicate that the silence behavior of OR nurses was predominantly characterized by other-oriented (prosocial) and self-protective (defensive) motivations rather than by passive compliance (acquiescent) or disengagement (indifferent), implying that OR nurses tended to withhold opinions out of consideration for team interests or fear of negative consequences, rather than from apathy or habitual conformity.

**TABLE 1:** Descriptive statistics for silence behavior, psychological capital, and job embeddedness (*N* = 395).

Variable	Items (n)	Min	Max	Mean ± SD	Score range	Item mean
Silence behavior						
Acquiescent silence	6	6	30	13.20 ± 4.40	6–30	2.20
Defensive silence	5	5	25	13.85 ± 4.30	5–25	2.77
Prosocial silence	5	5	25	14.45 ± 4.50	5–25	2.89
Indifferent silence	4	4	20	7.81 ± 2.95	4–20	1.95
Total	20	20	100	49.31 ± 15.83	20–100	2.47
Psychological capital						
Self-efficacy	6	6	36	27.50 ± 4.20	6–36	4.58
Hope	6	6	36	27.80 ± 4.50	6–36	4.63
Resilience	6	6	36	25.86 ± 4.10	6–36	4.31
Optimism	6	6	36	26.50 ± 4.00	6–36	4.42
Total	24	24	144	107.66 ± 16.81	24–144	4.49
Job embeddedness						
Organizational fit	7	7	35	23.10 ± 3.20	7–35	3.30
Community fit	6	6	30	19.50 ± 2.80	6–30	3.25
Organizational links	7	7	35	23.85 ± 3.50	7–35	3.41
Community links	5	5	25	16.50 ± 2.40	5–25	3.30
Organizational sacrifice	7	7	35	23.95 ± 3.30	7–35	3.42
Community sacrifice	5	5	25	18.81 ± 2.60	5–25	3.76
Total	37	37	185	125.71 ± 18.55	37–185	3.40

SD, standard deviation. The item mean represents the average score per item (total dimension score divided by the number of items), enabling direct comparison across dimensions of different lengths.

### Measurement model evaluation

Before testing the structural relationships among psychological capital, job embeddedness, and silence behavior, CFA was conducted to evaluate the measurement models of the three study scales. The Psychological Capital Questionnaire demonstrated acceptable fit for the hypothesized four-factor model (χ^2^/df = 2.74, RMSEA = 0.067, CFI = 0.912, TLI = 0.904), with all standardized factor loadings above 0.50. The Job Embeddedness Scale also showed acceptable fit for the six-factor model (χ^2^/df = 2.86, RMSEA = 0.071, CFI = 0.903, TLI = 0.896), and most standardized factor loadings exceeded 0.50. Similarly, the Nurse Organization Silence Assessment Questionnaire demonstrated acceptable fit for the four-factor model (χ^2^/df = 2.61, RMSEA = 0.064, CFI = 0.918, TLI = 0.909), with all standardized factor loadings above 0.50. These results supported the adequacy of the measurement models and justified the subsequent structural equation modeling analysis.

### Correlations among the three constructs

Pearson correlation analyses revealed that silence behavior was significantly negatively correlated with psychological capital (*r* = −0.556, *p* < 0.001) and with job embeddedness (*r* = −0.559, *p* < 0.001), while psychological capital was significantly positively correlated with job embeddedness (*r* = 0.621, *p* < 0.001). All three pairwise correlations supported Hypotheses H1–H3.

### Multiple linear regression analysis

To further identify factors associated with silence behavior among OR nurses, multiple linear regression analysis was conducted with silence behavior as the dependent variable. The total scores of psychological capital and job embeddedness, together with the demographic variables that were statistically significant in the univariate analyses, were entered simultaneously as independent variables. The subdimensions of psychological capital and job embeddedness were not entered together with their respective total scores, in order to reduce the risk of multicollinearity. The results showed that psychological capital, job embeddedness, professional title, employment type, and monthly income were significant predictors of silence behavior (*p* < 0.05). Multicollinearity diagnostics indicated that all tolerance values were above 0.20 and all VIF values were below 2 (range: 1.08–1.78), well within the acceptable thresholds, suggesting that multicollinearity did not materially distort the regression estimates (Table 2).

**TABLE 2:** Multiple linear regression analysis of predictors of silence behavior (*N* = 395).

Variable	B	SE	*t*	*p*	95% CI	β	Tolerance	VIF
Psychological capital (total)	−0.276	0.041	−6.73	<0.001	(−0.357, −0.195)	−0.293	0.58	1.72
Job embeddedness (total)	−0.295	0.045	−6.56	<0.001	(−0.383, −0.207)	−0.346	0.56	1.78
Gender	−1.910	1.130	−1.690	0.092	(−4.131, 0.311)	−0.058	0.93	1.08
Professional title	−2.348	0.752	−3.122	0.002	(−3.827, −0.869)	−0.127	0.81	1.23
Employment type	−1.985	0.730	−2.719	0.007	(−3.421, −0.549)	−0.087	0.85	1.18
Monthly income	−1.732	0.671	−2.581	0.010	(−3.051, −0.413)	−0.094	0.79	1.27

B, unstandardized regression coefficient; SE, standard error; β, standardized regression coefficient; 95% CI, 95% confidence interval for B; VIF, variance inflation factor. *R*^2^ = 0.487, Adjusted *R*^2^ = 0.479, *F*(6, 388) = 61.40, *p* < 0.001. All variables were entered simultaneously. All tolerance values were above 0.20 and all VIF values were below 2, indicating that multicollinearity was not a serious concern.

### Structural equation modeling and mediation analysis

**FIGURE 1:**
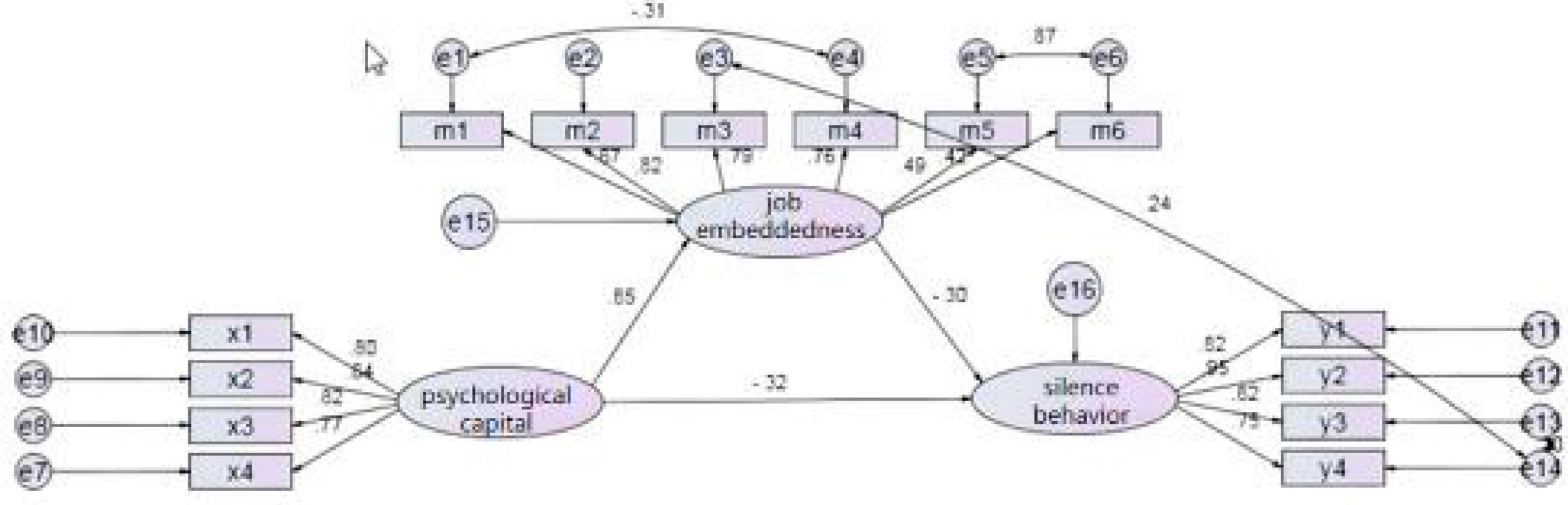
Structural equation model showing the relationships among psychological capital, job embeddedness, and silence behavior.

To further examine the relationships among psychological capital, job embeddedness, and silence behavior, a structural equation model was specified with psychological capital as the predictor, job embeddedness as the mediator, and silence behavior as the outcome (Figure 1). The model fit indices indicated a satisfactory fit (Table 3A). All hypothesized paths were statistically significant (Table 3B): psychological capital was positively associated with job embeddedness (β = 0.620, *p* < 0.001), job embeddedness was negatively associated with silence behavior (β = −0.348, *p* < 0.001), and psychological capital was directly negatively associated with silence behavior (β = −0.340, *p* < 0.001) after controlling for job embeddedness.

**TABLE 3A:** Structural equation model fit indices and standardized path coefficients (*N* = 395).

(A) Model fit indices							
**Model**	**χ^2^**	**df**	**χ^2^/df**	**CFI**	**TLI**	**RMSEA**	**SRMR**
Acceptable criteria	–	–	<3	>0.90	>0.90	<0.08	<0.08
Hypothesized model	186.43	83	2.246	0.945	0.932	0.055	0.048

**TABLE 3B:** Standardized path coefficients.

(B) Standardized path coefficients					
**Path**	**β**	**SE**	**z**	***P***	**95% CI**
Psychological capital → Job embeddedness	0.621	0.040	15.52	<0.001	(0.542, 0.698)
Psychological capital → Silence behavior	−0.340	0.052	−6.54	<0.001	(−0.442, −0.238)
Job embeddedness → Silence behavior	−0.348	0.050	−6.96	<0.001	(−0.446, −0.250)

CFI, Comparative Fit Index; TLI, Tucker–Lewis Index; RMSEA, Root Mean Square Error of Approximation; SRMR, Standardized Root Mean Square Residual; 95% CI = bias-corrected bootstrap confidence interval. Estimation method: maximum likelihood; bootstrap resamples = 5,000.

The mediation effect was estimated and tested using the bias-corrected bootstrap method with 5,000 resamples. The results indicated that job embeddedness played a significant mediating role in the relationship between psychological capital and silence behavior, suggesting a partial mediation pattern. The 95% confidence interval for the indirect effect was (−0.278, −0.158) and did not include zero. The indirect association of psychological capital with silence behavior through job embeddedness was −0.216, accounting for 38.85% of the total association (−0.556). Detailed results are presented in Table 4.

**TABLE 4:** Bootstrap mediation analysis: indirect effect of psychological capital on silence behavior via job embeddedness (*N* = 395).

Effect path	β	SE	z	*p*	95% CI LL	95% CI UL
Total effect (c): PC → SB	−0.556	0.044	−12.64	<0.001	−0.642	−0.470
Direct effect (c′): PC → SB (via JE)	−0.340	0.052	−6.54	<0.001	−0.442	−0.238
Indirect effect (a × b): PC → JE → SB	−0.216	0.030	–	–	−0.278	−0.158
Path a: PC → JE	0.621	0.040	15.52	<0.001	0.542	0.698
Path b: JE → SB	−0.348	0.050	−6.96	< 0.001	−0.446	−0.250

PC, psychological capital; JE, job embeddedness; SB, silence behavior; 95% CI, bias-corrected and accelerated (BCa) bootstrap confidence interval based on 5,000 bootstrap samples; LL, lower limit; UL, upper limit. The total effect equals the sum of the direct and indirect effects [−0.340 + (−0.216) = −0.556]; the indirect effect accounts for 38.85% of the total effect.

## Discussion

The results of this study revealed that OR nurses exhibited moderate levels of silence behavior. Among its dimensions, prosocial silence scored highest, followed by defensive, acquiescent, and indifferent silence. This pattern may be related to the leadership style of nursing managers, which has previously been associated with nurses’ silence tendencies. A lack of an open communication environment, together with a centralized management approach, may correspond to higher levels of silence behavior (34). In light of this, nursing leaders are encouraged to actively invite staff to voice their opinions, cultivate a supportive atmosphere for open dialogue, and incorporate input from all team members into decision-making processes (35).

This study found significant differences in silence behavior scores among OR nurses based on professional titles, employment status, and monthly income. Nurses with senior professional titles, longer OR hours, or higher monthly incomes tended to report lower levels of silence behavior. Compared with newly recruited nurses, more experienced nurses—who had longer clinical working hours, greater experience, higher incomes, and more opportunities for professional title advancement—often served as the backbone of the department, and were more likely to offer suggestions on work-related issues (36). Conversely, nurses with 1 year or less of experience faced challenges such as mastering various clinical nursing skills, acclimating to the working environment, and developing interpersonal relationships. Their limited engagement with departmental development was associated with lower participation in discussions and fewer contributions (37). Additionally, income was associated with nurses’ silence behavior; those with higher incomes tended to engage more actively in departmental development, a pattern that may relate to a stronger interest in departmental progress and the stability of their earnings.

This study identified a significant negative correlation between OR nurses’ psychological capital and silence behavior, indicating that nurses with higher psychological capital tended to report less silence behavior. These results were consistent with the findings of Kaya et al. (38), who reported that individuals with higher psychological capital displayed stronger motivation for knowledge exchange and extra-role behaviors. Such nurses tended to share tacit knowledge (e.g., experiential insights), reported higher communication competence, and cultivated broader interpersonal networks, which were in turn associated with greater professional influence. Their confidence and optimism have been linked to more innovative problem-solving and goal achievement (39). Similarly, job embeddedness showed a negative correlation with silence behavior. Nurses deeply embedded in their roles tended to prioritize organizational welfare, demonstrated loyalty, and reported stronger emotional attachment to their institution (40). They tended to perceive themselves as core organizational members, aligning personal growth with institutional success in accessing social and skill-based resources. In reciprocation, they tended to invest greater effort in their work and to voice constructive suggestions to address systemic issues. Lastly, this study also identified a positive correlation between psychological capital and job embeddedness among OR nurses. This was consistent with previous research findings (41). Psychological capital has been reported to be associated with greater job satisfaction and lower turnover intention among nurses (42). A positive psychological state may be associated with better role adaptation, higher subjective job satisfaction, greater enthusiasm for nursing activities, improved competency, and ultimately better nursing performance. Higher psychological capital may also correspond to greater satisfaction and identification with the team among OR nurses, which has previously been linked to better adaptability both within and outside of work and to lower turnover intention.

The findings of this study revealed that the psychological capital of OR nurses was not only directly associated with their silence behavior but was also indirectly associated with it through job embeddedness, with job embeddedness playing a partial mediating role in the relationship between psychological capital and silence behavior. Accordingly, a higher level of job embeddedness was associated with greater active participation in departmental development and lower levels of silence behavior, aligning with previous research findings (43). As a potential coping resource, psychological capital may be regarded as an important psychological asset associated with better coping with work stress, higher nursing work efficiency, and lower job burnout (37).

A positive psychological state may be associated with greater utilization of the hospital’s interpersonal network and resource platform, allowing nurses to accumulate psychological and material resources in their work and to report a stronger sense of belonging, loyalty, goal alignment, and pride toward the hospital and their department (44). This, in turn, may be associated with better compatibility between individuals and the organization in terms of goals, culture, and values. When nurses’ job embeddedness and psychological capital reached higher levels, their attachment and identification with the organization were more likely to be stronger, which may be associated with greater willingness to undertake behaviors beneficial to the development of their department (45).

Moreover, this group tended to report a stronger orientation toward the interests of the department, particularly when potential issues arose in the department’s development. Such nurses also appeared more attentive to these matters and more inclined to consider possible solutions and to offer related suggestions.

## Strength and limitation

To our knowledge, this study represents one of the first empirical investigations examining the interrelationships between psychological capital, silence behavior, and job embeddedness among Chinese operating room nurses, thereby contributing novel insights to the nursing behavior literature. However, several methodological limitations should be acknowledged. First, the exclusive reliance on self-reported measures may introduce response bias due to participants’ subjective perceptions. Moreover, because the independent, mediating, and dependent variables were all collected from the same respondents using the same self-report method, the possibility of common method bias cannot be entirely excluded. In addition, although the WeChat group served solely as a distribution channel and a passive technical support platform—with researchers responding only to participant-initiated procedural queries and having no access to individual responses—the mere presence of researchers in the group may be perceived as a form of intervention that could have influenced participants’ responses. Specifically, this researcher accessibility may have introduced a social desirability effect, potentially causing participants to underreport silence behavior due to a perceived sense of being observed. Several design features were implemented to mitigate this risk: the survey was fully anonymous, researchers could not link WeChat identities to questionnaire responses, no proactive contact or reminders were sent to individual participants, and no content-related guidance was provided at any point during the data collection period. Nevertheless, this potential confound cannot be completely excluded, and future studies should consider using fully automated, anonymous survey platforms without any researcher contact channel during data collection to further eliminate this limitation.

Furthermore, although several procedural controls (e.g., anonymous administration, varied scale formats, and non-disclosure of hypothesized relationships) and Harman’s single-factor test were employed to assess and mitigate common method bias, these measures have well-documented limitations and cannot entirely rule out its influence; therefore, the potential effect of CMB should still be considered when interpreting the results. In addition, although the regression model was specified using the total scores of psychological capital and job embeddedness to reduce the likelihood of multicollinearity. Multicollinearity was formally assessed using variance inflation factor (VIF) and tolerance values. All VIF values were below 2 and all tolerance values exceeded 0.20, indicating that multicollinearity was not a substantive concern in the present analysis. Second, the cross-sectional design precludes causal inferences about the observed relationships. Third, a non-probability convenience sampling strategy was adopted at the hospital level, and within each participating hospital all eligible OR nurses were invited rather than randomly sampled; because participants were drawn from only four tertiary hospitals in a single province in China, the generalizability of the findings to OR nurses in other regions, healthcare systems, or cultural contexts may be limited, and the results should therefore be interpreted with caution.

Future research should: (1) Incorporate objective behavioral measures alongside self-reports, (2) Employ longitudinal designs to establish temporal relationships, (3) Explore potential moderating factors through multi-center studies.

## Conclusion

This study found that operating room nurses showed moderate levels of silence behavior, psychological capital, and job embeddedness. Psychological capital was negatively associated with silence behavior, whereas job embeddedness was positively associated with psychological capital and negatively associated with silence behavior. In addition, job embeddedness played a partial mediating role in the association between psychological capital and silence behavior. These findings provide evidence of significant associations among psychological capital, job embeddedness, and silence behavior in operating room nurses.

## Data Availability

The raw data supporting the conclusions of this article will be made available by the authors, without undue reservation.

## References

[1] Aslan SK Yalçın B Göktepe N Türkmen E Canbolat S Bakoğlu N Serbest Ş. Effects of demographic, occupational, and practice environment variables on organizational silence among nurse managers. *Int Nurs Rev*. (2022) 69:132–8. 10.1111/inr.12712 34480355

[2] Zhou Y Li X. Effect assessment of the application value of evidence-based nursing intervention in operating room nursing: a protocol for a systematic review and meta-analysis. *Medicine.* (2021) 100:e26867. 10.1097/MD.0000000000026867 34397899 PMC8360402

[3] Lappeman M Swartz L. Rethinking obstetric violence and the “neglect of neglect”: the silence of a labour ward milieu in a South African district hospital. *BMC Int Health Hum Rights*. (2019) 19:30. 10.1186/s12914-019-0218-2 31666133 PMC6822362

[4] Sutcliffe KM Lewton E Rosenthal MM. Communication failures: an insidious contributor to medical mishaps. *Acad Med.* (2004) 79:186–94. 10.1097/00001888-200402000-00019 14744724

[5] Zhao X Shi C Zhao L. Nurses’ intentions, awareness and barriers in reporting adverse events: a cross-sectional survey in tertiary hospitals in China. *Risk Manage Healthc Policy.* (2022) 15:1987–97. 10.2147/RMHP.S386458 36329826 PMC9624208

[6] Morrison EW Milliken FJ. Organizational silence: a barrier to change and development in a pluralistic world. *Acad Manage Rev.* (2000) 25:706–25. 10.2307/259200

[7] Rosen S Tesser A. On reluctance to communicate undesirable information: the MUM effect. *Sociometry.* (1970) 33:253–253. 10.2307/2786156 31737403 PMC6815610

[8] Dyne LV Ang S Botero IC. Conceptualizing employee silence and employee voice as multidimensional constructs. *J Manage Stud.* (2003) 40:1359–92. 10.1111/1467-6486.00384

[9] De Los Santos JAA Rosales RA Falguera CC Firmo CN Tsaras K Labrague LJ. Impact of organizational silence and favoritism on nurse’s work outcomes and psychological well-being. *Nurs Forum*. (2020) 55:782–92. 10.1111/nuf.12496 32794250

[10] Moloney W Fieldes J Jacobs S. An integrative review of how healthcare organizations can support hospital nurses to thrive at work. *Int J Environ Res Public Health*. (2020) 17:8757. 10.3390/ijerph17238757 33255725 PMC7728312

[11] Kritsotakis G Gkorezis P Andreadaki E Theodoropoulou M Grigoriou G Alvizou A et al. Nursing practice environment and employee silence about patient safety: the mediating role of professional discrimination experienced by nurses. *J Adv Nurs*. (2022) 78:434–45. 10.1111/jan.14994 34337760

[12] Lee SE Seo JK Squires A. Voice, silence, perceived impact, psychological safety, and burnout among nurses: a structural equation modeling analysis. *Int J Nurs Stud*. (2024) 151:104669. 10.1016/j.ijnurstu.2023.104669 38160639

[13] She J Zhang R Li Y Mei Y Li H. Effect of ethical leadership on nurses’ organizational silence: the mediating role of organizational justice. *J Nurs Manag*. (2023) 2023:9929435. 10.1155/2023/9929435 40225636 PMC11918815

[14] Zheng XT Ke JL Shi JT Zheng XS. Survey on employee silence and the impact of trust on it in China. *Acta Psychol Sin.* (2008) 40:219–27. 10.3724/SP.J.1041.2008.00219

[15] Yurdakul M Beşen MA Erdoğan S. The organisational silence of midwives and nurses: reasons and results. *J Nurs Manag*. (2016) 24:686–94. 10.1111/jonm.12374 27040073

[16] Elliott R Fry M. Psychological capital, well-being, and patient safety attitudes of nurses and midwives: a cross-sectional survey. *Nurs Health Sci*. (2021) 23:237–44. 10.1111/nhs.12808 33382147

[17] De Los Santos JAA Labrague LJ. Job engagement and satisfaction are associated with nurse caring behaviours: a cross-sectional study. *J Nurs Manag*. (2021) 29:2234–42. 10.1111/jonm.13384 34021940

[18] Waltz LA Muñoz L Weber Johnson H Rodriguez T. Exploring job satisfaction and workplace engagement in millennial nurses. *J Nurs Manag*. (2020) 28:673–81. 10.1111/jonm.12981 32068932

[19] Henriksen K Dayton E. Organizational silence and hidden threats to patient safety. *Health Serv Res*. (2006) 41:1539–54. 10.1111/j.1475-6773.2006.00564.x 16898978 PMC1955340

[20] Saadi A Taleghani S Dillard A Ryan G Heilemann M Eisenman D. Original research: nurses’ experiences with racial, ethnic, cultural, and religious discrimination in the workplace: a qualitative study. *Am J Nurs*. (2023) 123:24–34. 10.1097/01.NAJ.0000931892.39368.e1 37021974

[21] Harmanci Seren AK Topcu I Eskin Bacaksiz F Unaldi Baydin N Tokgoz Ekici E Yildirim A. Organisational silence among nurses and physicians in public hospitals. *J Clin Nurs*. (2018) 27:1440–51. 10.1111/jocn.14294 29399900

[22] Gkorezis P Panagiotou M Theodorou M. Workplace ostracism and employee silence in nursing: the mediating role of organizational identification. *J Adv Nurs*. (2016) 72:2381–8. 10.1111/jan.12992 27113971

[23] Wang Y. *Research on the Correlation between the Multiple Leadership Styles of Head Nurses in Operating Rooms and Nurses’ Silent Behaviors and Work Engagement.* Changchun: Jilin University (2017).

[24] Luthans F Avolio BJ Avey JB Norman SM. Positive psychological capital: measurement and relationship with performance and satisfaction. *Pers. Psychol.* (2007) 60:541–72. 10.1111/j.1744-6570.2007.00083.x

[25] Hobfoll SE. Conservation of resources. A new attempt at conceptualizing stress. *Am Psychol*. (1989) 44:513–24. 10.1037//0003-066x.44.3.513 2648906

[26] Mitchell TR Holtom BC Lee TW Sablynski CJ Erez M. Why people stay: using job embeddedness to predict voluntary turnover. *Acad Manage J.* (2001) 44:1102–21. 10.2307/3069391

[27] Blau PM. *Exchange and Power in Social Life.* New York, NY: John Wiley & Sons (1964).

[28] Kendall MG. *Rank Correlation Methods.* London: Charles Griffin (1975).

[29] Kline RB. *Principles and Practice of Structural Equation Modeling.* 4th ed. New York, NY: The Guilford Press (2016).

[30] Hu L Bentler PM. Cutoff criteria for fit indexes in covariance structure analysis: conventional criteria versus new alternatives. *Struct Equat Model.* (1999) 6:1–55. 10.1080/10705519909540118

[31] Ding Y Yang Y Yang X Zhang T Qiu X He X et al. The mediating role of coping style in the relationship between psychological capital and burnout among chinese nurses. *PLoS One*. (2015) 10:e0122128. 10.1371/journal.pone.0122128 25898257 PMC4405204

[32] Yang J. *Preliminary Development of the Questionnaire for assessing Nurses’ Silent Behaviors.* Taiyuan: Shanxi Medical University (2016).

[33] Podsakoff PM Mackenzie SB Lee JY Podsakoff NP. Common method biases in behavioral research: a critical review of the literature and recommended remedies. *J Appl Psychol.* (2003) 88:879–903. 10.1037/0021-9010.88.5.879 14516251

[34] Alhojairi HM Elseesy NAM Mahran SM Banakhar MA Alsharif F. Assessment of nurses’ workplace silence behaviour motives: a cross-sectional study. *Int J Nurs Sci*. (2024) 11:553–62. 10.1016/j.ijnss.2024.10.006 39698129 PMC11650691

[35] Erkutlu H Chafra J. Leader’s integrity and employee silence in healthcare organizations. *Leadersh Health Serv*. (2019) 32:419–34. 10.1108/LHS-03-2018-0021 31298087

[36] Labrague LJ De Los Santos JA. Association between nurse and hospital characteristics and organisational silence behaviours in nurses: a cross-sectional study. *J Nurs Manag*. (2020) 28:2196–204. 10.1111/jonm.13101 32668491

[37] Yuan Z Zhang X Wang F Jin M Teng M He H et al. Levels of psychological capital among nurses: a systematic review and meta-analysis. *Int Nurs Rev*. (2023) 70:89–96. 10.1111/inr.12803 36205604

[38] Kaya G Eskin Bacaksiz F. The relationships between nurses’ positive psychological capital, and their employee voice and organizational silence behaviors. *Perspect Psychiatr Care*. (2022) 58:1793–800. 10.1111/ppc.12990 34888883

[39] Liu Y Aungsuroch Y Gunawan J Zeng D. Job stress, psychological capital, perceived social support, and occupational burnout among hospital nurses. *J Nurs Scholarsh*. (2021) 53:511–8. 10.1111/jnu.12642 33646610

[40] Fan S Zhou S Ma J An W Wang H Xiao T. The role of the nursing work environment, head nurse leadership and presenteeism in job embeddedness among new nurses: a cross-sectional multicentre study. *BMC Nurs*. (2024) 23:159. 10.1186/s12912-024-01823-1 38443951 PMC10913553

[41] Chen H Kewou NYN Atingabili S Sogbo ADZ Tcheudjeu AT. The impact of psychological capital on nurses’ job performance: a chain mediation analysis of problem-focused coping and job engagement. *BMC Nurs*. (2024) 23:149. 10.1186/s12912-024-01802-6 38431587 PMC10909282

[42] Da S He Y Zhang X. Effectiveness of psychological capital intervention and its influence on work-related attitudes: daily online self-learning method and randomized controlled trial design. *Int J Environ Res Public Health*. (2020) 17:8754. 10.3390/ijerph17238754 33255704 PMC7728090

[43] Song R Sun N Song X. The efficacy of psychological capital intervention (PCI) for depression from the perspective of positive psychology: a pilot study. *Front Psychol*. (2019) 10:1816. 10.3389/fpsyg.2019.01816 31447745 PMC6692487

[44] Xia M Wang J Wang Z Bi D Mao H Liu X et al. Examining the relationship between nurse psychological capital and job burnout: a multilevel analysis across nurse, nurse leader, and nurse family perspectives. *Hum Resour Health*. (2025) 23:18. 10.1186/s12960-025-00986-5 40155900 PMC11951840

[45] Alan H Polat S Tiryaki Sen H. The role of psychological capital in the relationship between nurses’ job satisfaction and turnover intention. *Perspect Psychiatr Care*. (2022) 58:2811–9. 10.1111/ppc.13128 35726709

